# Upregulation of basolateral small conductance potassium channels (KCNQ1/KCNE3) in ulcerative colitis

**DOI:** 10.1016/j.bbrc.2015.12.086

**Published:** 2016-02-05

**Authors:** Adel Al-Hazza, John Linley, Qadeer Aziz, Malcolm Hunter, Geoffrey Sandle

**Affiliations:** a Institute of Membrane and Systems Biology, University of Leeds, UK; b Leeds Institute of Biomedical and Clinical Sciences, St. James's University Hospital, Leeds, UK

**Keywords:** Human colonic crypts, Ulcerative colitis, KCNQ1/KCNE3 channels, Patch clamp

## Abstract

**Background:**

Basolateral K^+^ channels hyperpolarize colonocytes to ensure Na^+^ (and thus water) absorption. Small conductance basolateral (KCNQ1/KCNE3) K^+^ channels have never been evaluated in human colon. We therefore evaluated KCNQ1/KCNE3 channels in distal colonic crypts obtained from normal and active ulcerative colitis (UC) patients.

**Methods:**

KCNQ1 and KCNE3 mRNA levels were determined by qPCR, and KCNQ1/KCNE3 channel activity in normal and UC crypts, and the effects of forskolin (activator of adenylate cyclase) and UC-related proinflammatory cytokines on normal crypts, studied by patch clamp recording.

**Results:**

Whereas KCNQ1 and KCNE3 mRNA expression was similar in normal and UC crypts, single 6.8 pS channels were seen in 36% of basolateral patches in normal crypts, and to an even greater extent (74% of patches, P < 0.001) in UC crypts, with two or more channels per patch. Channel activity was 10-fold higher (P < 0.001) in UC crypts, with a greater contribution to basolateral conductance (5.85 ± 0.62 mS cm^−2^) than in controls (0.28 ± 0.04 mS cm^−2^, P < 0.001). In control crypts, forskolin and thromboxane A_2_ stimulated channel activity 30-fold and 10-fold respectively, while PGE_2_, IL-1β, and LTD_4_ had no effect.

**Conclusions:**

KCNQ1/KCNE3 channels make only a small contribution to basolateral conductance in normal colonic crypts, with increased channel activity in UC appearing insufficient to prevent colonic cell depolarization in this disease. This supports the proposal that defective Na^+^ absorption rather than enhanced Cl^−^ secretion, is the dominant pathophysiological mechanism of diarrhea in UC.

## 1 Introduction

Basolateral potassium (K^+^) channels play a central role in maintaining colonic epithelial cells in a hyperpolarized state, which is a critical requirement for ionic movements across the apical and basolateral membranes. In healthy human colonic epithelium, the basolateral K^+^ conductance largely reflects intermediate conductance basolateral K^+^ (IK; KCNN4) channels [1]. In active ulcerative colitis (UC), the marked decrease in IK channel expression/activity (with ensuing cellular depolarization) has been proposed as one of the factors contributing to colonic malabsorption of Na^+^ (and thus water) in this disease [1]. Indeed, diarrhea, the dominant symptom in UC, largely reflects defective Na^+^ and water absorption, rather than increased Cl^−^ secretion across the inflamed colonic mucosa [2], [3], [4], [5].

Although IK channels dominate the basolateral K^+^ conductance in human colon [1]. basolateral membranes of mouse small intestinal and colonic crypt cells also contain small conductance K^+^ (SK or KCNQ1/KCNE3) channels [6], [7], [8]. However, the possible existence of KCNQ1/KCNE3 channels and their contribution to the basolateral K^+^ conductance of human colonic crypt cells, has never been explored. KCNQ1 is a K^+^ channel protein with six transmembrane domains, and interacts physically with the smaller β-subunit KCNE3 protein, which has a single transmembrane domain [9]. KCNE3 changes the biophysical properties of KCNQ1, the KCNQ1/KCNE3 complex forming a constitutively open basolateral K^+^ channel, which is present in mammalian small intestine and colon, is readily stimulated by cAMP, markedly inhibited by chromanol 293B, and is a prerequisite for cAMP-stimulated electrogenic Cl^−^ secretion, such as that triggered by a variety of intestinal pathogens [9]. The role of KCNQ1/KCNE3 channels appears to be to maintain the Cl^−^ secretory response by recycling K^+^ entering the cell via the basolateral Na^+^–K^+^–2Cl^−^ cotransporter, thus hyperpolarizing the cell while stimulating basolateral Cl^−^ uptake as a prelude to apical Cl^−^ exit via CFTR Cl^−^ channels [9]. However, the transmucosal electrical properties of the colon in active UC are not consistent with electrogenic Cl^−^ secretion [2], [3], nor has Cl^−^ secretion been reported in this disease in previous ion transport studies [2], [10], which raises the possibility that the expression/activity of putative KCNQ1/KCNE3 channels in the inflamed colon might be decreased. The aims of the present study were to identify and characterize KCNQ1/KCNE3 channels in healthy (control) human colon, and then study their activity in patients with active UC.

## 2 Materials and methods

### 2.1 Quantitative polymerase chain reaction (qPCR)

Crypts for qPCR were isolated from 5 to 6 sigmoid mucosal biopsies taken during colonoscopic evaluation of patients with functional abdominal pain who were free of inflammatory bowel disease (controls, n = 9), and patients with moderately active UC (n = 7), by Ca^2+^ chelation [11]. Crypts were pelleted by centrifugation and total RNA extracted using TRI reagent (Sigma) according to the manufacturer's instructions. RNA was quantified by a RiboGreen (Invitrogen, Paisley, UK) assay, using a standard curve constructed with RNA of known concentrations. 1 μg of total RNA was reverse transcribed at 37 °C using Superscript reverse transcriptase (Invitrogen, Paisley, UK) with oligo-(dT)_12–18_ primers according to the manufacturer's instructions.

cDNA was subsequently used as a template for 40 rounds of PCR with intron-spanning primers specific for human KCNQ1 (sense AGCAGAAGCAGAGGCAGAAG; antisense CTGGGTGACAGCAGAGTGTG, KCNE3 (sense GCAGAGGGAAATGAGACAGG; antisense CAGGGTGGTGGTGGTAAATC) and β-actin (sense AGAAAATCTGGCACCACACC; antisense GGGTGTTGAAGGTCTCAAA), the latter acting as an endogenous reference to allow compensation for small variations in sample concentration. Amplification of DNA products was measured in real-time (Lightcycler, Roche) by incorporating the DNA-binding dye SYBR Green I (Quantitech). Primer efficacy was validated by gel electrophoresis of the PCR reaction, and amplification of single products of the expected molecular weight (KCNQ1 160 bp; KCNE3 119 bp; β-actin 148 bp) confirmed by sequencing (Lark Technologies). Real-time PCR crossing point (Cp) was determined using the ‘second derivative maximum method’ [12]. PCR efficiency (E) was calculated from the slope of the log-linear amplification phase using the LinReg software package [13]. The amounts of KCNQ1 and KCNE3 mRNA relative to β-actin were expressed as E^Cp^_β-actin_/E^Cp^_channel_ [14]. The specificity of RT–PCR products was also routinely confirmed by melting curve analysis.

### 2.2 Patch clamp recording

Crypts for patch clamp studies were isolated from colonoscopic mucosal biopsies taken during evaluation of consented control patients (n = 34), and patients with moderately active UC (n = 9), by Ca^2+^ chelation [11]. None of the patients was receiving steroid treatment. Crypts were maintained on ice until required. Crypts were placed on a glass coverslip pre-treated with poly-l-lysine (0.1%) in a perfusion chamber (Warner Instruments), which was mounted on an inverted microscope (Nikon Diaphot). The perfusion chamber solution contained 140 mM NaCl, 4.5 mM KCl, 1.2 mM CaCl_2_, 1.2 mM MgCl_2_, 10 mM HEPES, and 5 mM glucose, titrated to pH 7.4 with NaOH. Patch pipettes were fabricated from soda-lime microhematocrit glass using a two-stage puller (Narishige, Model PP-83) and had tip resistances of 2–4 MΩ when filled with a solution containing 145 mM KCl, 1.2 mM CaCl_2_, 1.2 mM MgCl_2_, 10 mM HEPES and 5 mM glucose, titrated to pH 7.4 with KOH. After filling with solution, pipettes were subsequently dipped briefly, up to the base of the shank, in silane (Sigmacote, Sigma) to reduce stray capacitance. Patch clamp recordings were obtained in the cell-attached configuration from the basolateral membranes of cells in the mid-third region of crypts. Cell currents were amplified and low-pass filtered at 2 kHz (Axopatch 200B, Axon Instruments), and sampled to computer at 5 kHz via a Digidata 1320A interface (Axon Instruments). Command potentials (V_com_) were applied to the pipette interior and referenced to the bath (ground). Data acquisition and analysis were performed using pClamp 9.0 software (Axon Instruments). Channel activity was measured as N.P_o_, where P_o_ is the single channel open probability and N is the number of channels in the patch (taken to be the maximum number of open channels during the recording).

Having identified basolateral KCNQ1/KCNE3 channels in crypts from control patients, additional experiments were performed to determine the effect of 10 μM forskolin (an activator of adenylate cyclase, thereby increasing intracellular cAMP concentration), and a variety of inflammatory mediators present at increased concentration in the colonic epithelium of patients with active UC: 100 nM prostaglandin E_2_ (PGE_2_), 1 μM thromboxane A2 (TXA_2_), 10 nM interleukin 1-β (IL-1β), and 1 μM leukotriene D_4_ [15], [16], [17], [18]. Recordings of KCNQ1/KCNE3 channel activity were made before and 5 min after addition of the agent under study to the bath solution.

### 2.3 Statistics

Data are given as mean ± SEM, where n corresponds to the number of subjects unless otherwise indicated. Comparison between groups was made using the Wilcoxon Signed Rank test and Mann–Whitney U test for paired and unpaired data, respectively.

## 3 Results

### 3.1 Molecular characteristics of KCNQ1/KCNE3 channels in human colon

**Fig. 1:**
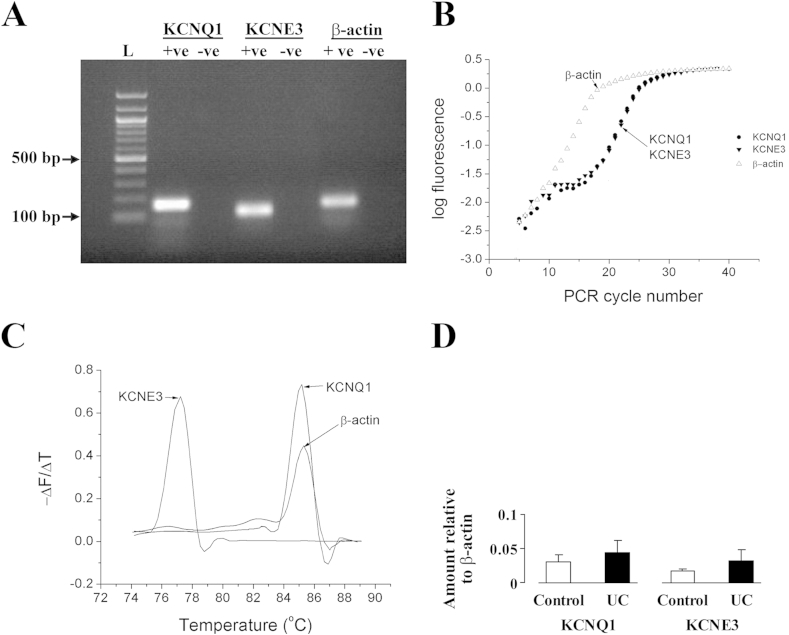
(A) Agarose gel showing RT-PCR products of KCNQ1 and KCNE3 in control human sigmoid colon. L = 100 bp ladder, +ve = cDNA template present, -ve = cDNA template absent. (B) Plot of logarithmic fluorescence versus RT-PCR cycle number, showing the log-linear phase of PCR amplification. (C) Melting curve analysis of PCR products. (D) Relative levels of KCNQ1 and KCNE3 mRNA in control and UC patients.

RT-PCR using specific primers indicated the presence of KCNQ1, KCNE3 and β-actin (as the internal control) in colonic crypt cells from control patients and those with active UC. Agarose gel (1.3%) electrophoresis showed that the single product bands were of the expected size (Fig. 1A), fragment sizes for KCNQ1, KCNE3 and β-actin being 160 bp, 119 bp and 148 bp, respectively. The identity of the products was confirmed by automated sequencing (Lark Technologies). Amplification of KCNQ1, KCNE3 and β-actin mRNAs was monitored using SYBR Green 1, and during PCR amplification, SYBR green fluorescence increased in a log-linear fashion once it was above the threshold for detection by the lightcycler (Fig. 1B). Specificity of the RT-PCR products was also confirmed by melting curve analysis, the rate of release of the fluorophore during DNA strand separation being greatest at the product-specific melting temperature (Tm), the presence of a single peak indicating the presence of a single product. The measured values of Tm for KCNQ1, KCNE3 and β-actin were 85.1 °C, 77.1 °C and 85.2 °C, respectively (Fig. 1C). There were no significant differences in the expression of KCNQ1 mRNA or KCNE3 mRNA between control patients and those with active UC (Fig. 1D).

### 3.2 Electrophysiological characteristics of KCNQ1/KCNE3 channels in human colon

**Fig. 2:**
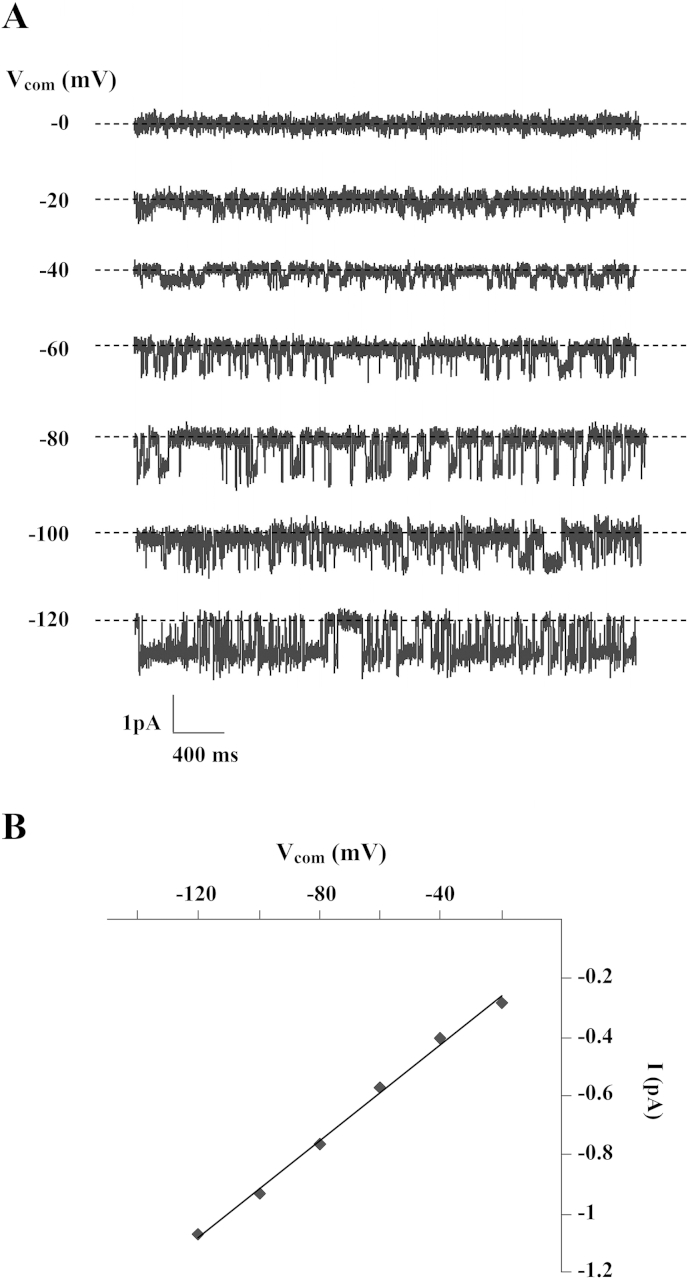
(A) SK channel currents across a cell-attached basolateral membrane patch on a crypt from control sigmoid colon at different command potentials (V_com_) referenced to the pipette interior. Broken line indicates closed channel current and downward deflections indicate channel openings. (B) Linear I/V_com_ relationship of the SK channel shown in A.

**Fig. 3:**
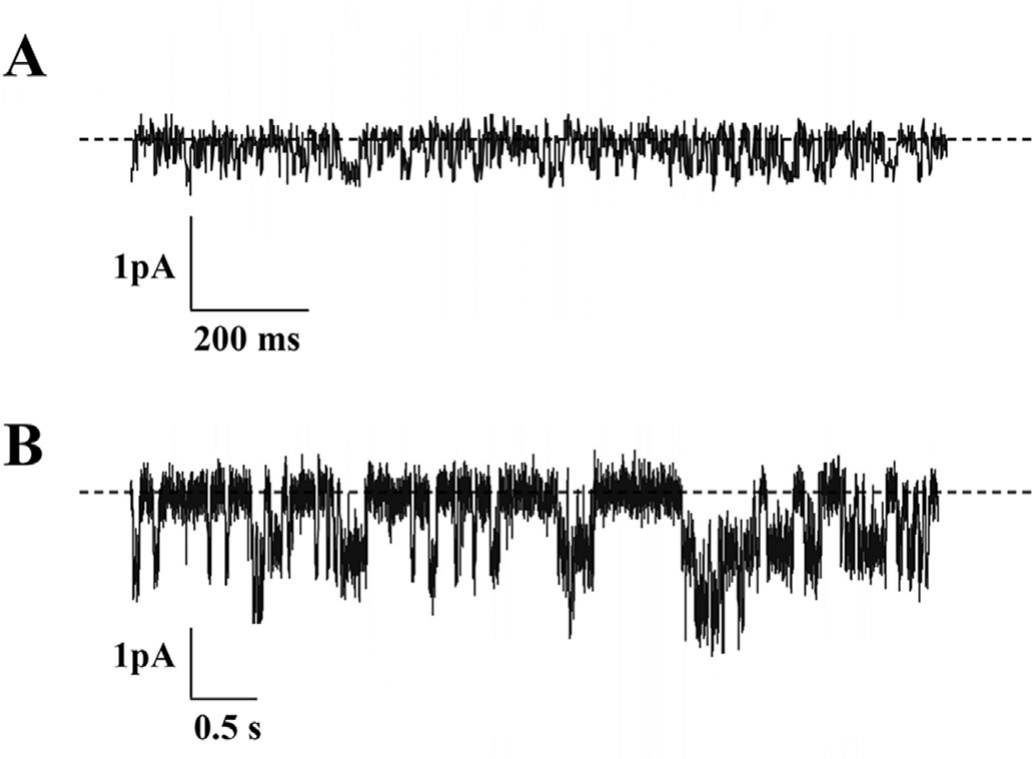
SK channel currents across cell-attached basolateral membrane patches on crypts from (A) control and (B) UC sigmoid colon, at a command potential (V_com_) of −100 mV (referenced to pipette interior). Broken line indicates closed channel current and downward deflections indicate channel openings.

KCNQ1/KCNE3 channel activity was studied in cell-attached patches of the basolateral membrane of cells in the mid-crypt region of crypts obtained from control patients (n = 34), channel activity being observed in 36% of patches. Representative recordings, together with the corresponding current–voltage (I/V_com_) relationship, are shown in Fig. 2A and B, respectively. Patches usually contained one channel (Fig. 2A), with a slope conductance of 6.8 ± 0.5 pS. This value was of the same order as those obtained for the basolateral KCNQ1/KCNE3 channel in rat colon crypt cells (∼3 pS) [19], and the human KCNQ1/KCNE3 channel (4.5 pS) expressed in *Xenopus* oocytes [20]. By contrast, KCNQ1/KCNE3 channel activity was observed in 74% of patches in crypts obtained from patients with active UC (n = 9), the difference in channel prevalence between the two groups of patients being significant (P < 0.001). Furthermore, patches from UC patients usually contained two or more channels (Fig. 3B), and overall KCNQ1/KCNE3 channel activity (*N*.*P*_o_) was 10-fold greater in UC patients (1.13 ± 0.28) than in control patients (0.11 ± 0.02, P < 0.001).

The physiological importance of the basolateral K^+^ conductance of epithelial cells in general lies in its role in allowing K^+^ ions to recycle across the membrane, thus keeping cells hyperpolarized, a prerequisite for vectorial ion transport. Based on the assumption that IK channels account for most of the basolateral K^+^ conductance in normal human colonic crypt cells, we previously reported that the decreases in basolateral IK channel expression and activity seen in active UC lead to a ∼75% decrease in basolateral K^+^ conductance [1]. In the present study, we calculated basolateral membrane conductance due to KCNQ1/KCNE3 channels (G_SK_) in control and UC patients as:

$${\text{G}}_{\text{SK}}=\text{fraction}\;\text{of}\;\text{patches}\;\text{containing}\;\text{channels}\times N.P_{\text{o}}\times g$$

with *N*.*P*_o_ taken as the average *N*.*P*_o_ of all patches obtained from an individual patient rather than the *N*.*P*_o_ of a single patch, a single channel conductance (*g*) of 7 pS, and an assumed patch area of 1 μm^2^. The calculated value of G_SK_ in patients with active UC (5.85 ± 0.62 mS cm^−2^) was significantly greater than the value in control patients (0.28 ± 0.04 mS cm^−2^, P < 0.001).

### 3.3 Activation of KCNQ1/KCNE3 channels in human colon

**Fig. 4:**
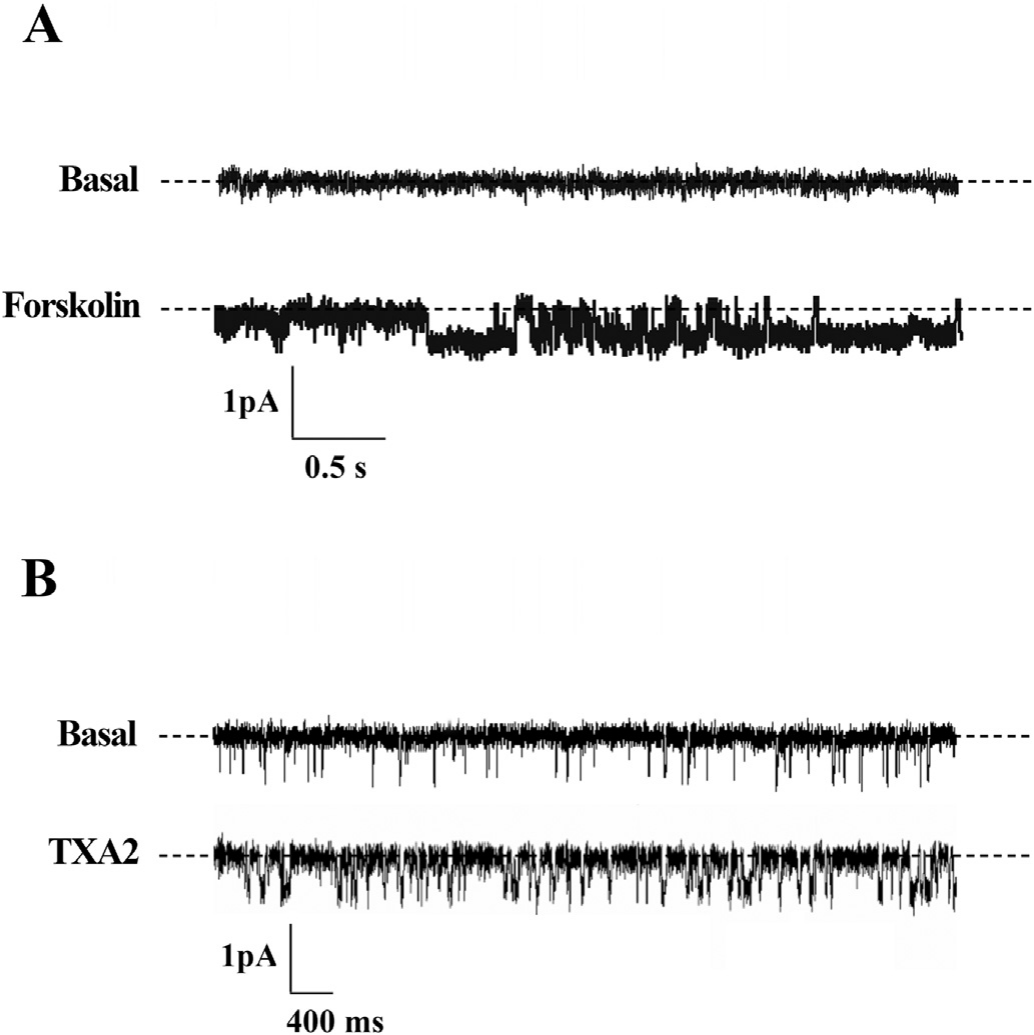
SK channel currents across cell-attached basolateral membrane patches on crypts from normal sigmoid colon, in the basal state and after the addition of 10 μM forskolin (A) or (B) 1 μM TXA_2_. Recordings were obtained at a command potential (V_com_) of −100 mV (referenced to pipette interior), broken lines indicating closed channel currents and downward deflections indicating channel openings.

In cell-attached patches using crypts from control patients, addition of 10 μM forskolin to the bath solution resulted in the stimulation of basolateral KCNQ1/KCNE3 channels, *N*.*P*_o_ increasing from 0.01 ± 0.003 to 0.32 ± 0.08 (P < 0.005, n = 8), and a representative experiment is shown in Fig. 4A. The addition of 1 μM TXA_2_ also stimulated KCNQ1/KCNE3 channel activity, *N*.*P*_o_ increasing from 0.02 ± 0.01 to 0.18 ± 0.07 (P < 0.05, n = 5), and a representative experiment is shown in Fig. 4B. However, neither 100 nM PGE_2_, 10 nM IL-1β, nor 1 μM LTD_4_ had any significant effect on KCNQ1/KCNE3 channel activity (0.08 ± 0.05 versus 0.24 ± 0.19, P = 0.4, n = 6; 0.12 ± 0.11 versus 0.28 ± 0.09, P = 0.25, n = 5; and 0.25 ± 0.18 versus 0.25 ± 0.08, P = 0.99, n = 4, respectively).

## 4 Discussion

The present study shows for the first time that (i) both KCNQ1 mRNA and KCNE3 mRNA are present in control human colonic epithelial cells, (ii) low basal levels of KCNQ1/KCNE3 channel activity are present in the basolateral membranes of these cells, and (iii) these low basal levels of KCNQ1/KCNE3 channel activity are markedly stimulated by forskolin-induced cAMP-dependent phosphorylation. We found the single channel conductance of basolateral KCNQ1/KCNE3 channels in control human colon (6.8 ± 0.5 pS) to be similar to that of human KCNQ1/KCNE3 channels expressed in *Xenopus* oocytes (4.5 pS) [20]. Based on the findings that basolateral KCNQ1/KCNE3 channels (i) co-exist with apical Cl^−^ (CFTR) channels in intestinal crypt cells [21], (ii) are stimulated by cAMP [8], and (iii) inhibited by chromanol 293B (resulting in the inhibition of electrogenic Cl^−^ secretion) [8], it has become clear that these K^+^ channels play a critical role during cAMP-activated Cl^−^ secretion, recycling K^+^ ions across the basolateral membrane to maintain a favourable electrical gradient for apical Cl^−^ exit, thus making them a prime target for novel anti-diarrheal drugs.

Although we observed an increase in basolateral KCNQ1/KCNE3 channel activity, and what appeared to be an increase in the number (and presumably density) of channels per patch in cells from UC patients compared with controls, the levels of KCNQ1 mRNA and KCNE3 mRNA were similar in the two groups. This suggests that the apparent increase in KCNQ1/KCNE3 channel density reflects a post-transcriptional event, possibly increased sorting of channel protein(s) to the basolateral membrane. This would be in broad agreement with our previous finding of uniform expression of high conductance apical potassium (BK; KCNMA1) channels along the entire surface-crypt cell axis in UC patients, whereas they are largely restricted to surface cells and cells in the upper 20% of crypts in control human colon [22]. Why KCNQ1/KCNE3 channel expression (or for that matter BK channel expression) should be increased in human UC is unclear, but it is interesting that butyrate, a key energy source for colonocytes, suppresses NKCC1 (basolateral Na^+^-K^+^–2Cl^−^ cotransporter isoform-1) gene expression in human colon-derived HT29 cells [23]. Thus, it is conceivable that reduced oxidation of butyrate by colonic epithelial cells, as occurs in UC [24], might result in upregulation of KCNQ1/KCNE3 and/or BK channels in this disease. In any event, our finding of increased basolateral KCNQ1/KCNE3 channel activity in active UC is in marked contrast to our previously reported observation of substantial decreases in basolateral IK channel expression and activity in these patients [1]. We have proposed that these changes may explain the cell depolarization seen in active UC, thereby decreasing the electrical driving force for electrogenic Na^+^ absorption (and as a consequence, Cl^−^ and water absorption) across the inflamed mucosa [1]. Despite the fact that the increase in basolateral KCNQ1/KCNE3 channel activity in UC patients resulted in a significantly greater calculated basolateral membrane conductance (G_SK_, 5.85 ± 0.62 mS cm^−2^) compared with controls (0.28 ± 0.04 mS cm^−2^, P < 0.001), this increase in G_SK_ was too small to fully compensate for the ∼17 mS cm^−2^ decrease in basolateral membrane conductance secondary to reduced IK channel expression and activity in UC patients [1]. Thus, while the increase in basolateral KCNQ1/KCNE3 channel activity in UC patients could be construed as an attempt to maintain colonocytes in a state of hyperpolarization, this clearly does not occur, as colonocytes in UC patients have been shown to be significantly depolarized in studies using intracellular microelectrodes [3].

Our studies using crypts from control patients showed that increasing intracellular cAMP (using exogenous forskolin) elicited a 30-fold rise in basolateral KCNQ1/KCNE3 channel activity. This observation is consistent with the chromanol 293B-inhibitable, cAMP-stimulated Cl^−^ secretion previously demonstrated in mouse colon [25], highlighting the channel's role in basolateral K^+^ recycling in order to maintain a favourable electrical gradient for apical Cl^−^ exit. PGE_2_ is an eicosanoid inflammatory mediator produced in leukocytes by the oxidation of arachidonic acid through arachidonate 5-lipoxygenase activity, and its intramucosal levels are substantially increased in UC [16]. Since the ion transport effects of PGE_2_ are at least partly mediated by cAMP [26], [27], the higher levels of KCNQ1/KCNE3 channel activity seen in UC patients may have reflected a cAMP-dependent stimulatory effect of PGE_2_. However, for reasons that are unclear, 100 nM PGE_2_ failed to activate KCNQ1/KCNE3 channel activity in control crypts. We also studied the effect of TXA_2_, another eicosanoid inflammatory mediator present at increased concentrations in the inflamed colonic mucosa in active UC, and capable of stimulating Cl^−^ secretion in normal rat colon [16]. Unlike PGE_2_, TXA_2_ elicited a 10-fold increase in KCNQ1/KCNE3 channel activity in control crypts, which suggests a regulatory link between TXA_2_ receptors and KCNQ1/KCNE3 channels. TxA_2_ has been shown to increase intracellular Ca^2+^ in platelets [28], and thus its stimulatory effect on KCNQ1/KCNE3 channels in human colonic crypts may also be Ca^2+^-mediated, since carbachol (a Ca^2+^-mediated cholinergic agonist) activated KCNQ1/KCNE3 channels during its stimulation of Cl^−^ secretion in mouse colon [29]. Intracellular Ca^2+^ regulates KCNQ1 via calmodulin and other Ca^2+^-sensor proteins [30]. Interestingly, the antidiarrheal drug loperamide markedly inhibits TxA_2_-induced Cl^−^ secretion in normal rat distal colon without affecting the TxA_2_-induced increase in intracellular Ca^2+^ concentration [31]. Furthermore, the specific calmodulin inhibitor W-7 has an even greater inhibitory effect on TxA_2_-induced Cl^−^ secretion, which suggests that loperamide's antisecretory effect involves blockade of the calmodulin system [31]. However, even if PGE_2_ and TXA_2_ stimulate KCNQ1/KCNE3 channel activity in active UC, this is not accompanied by an overall Cl^−^ secretory response, as net colonic Cl^−^ secretion does not occur in patients with this disease [2], [3]. By contrast, the proinflammatory mediators IL-1β (which originates from helper CD4 T lymphocytes, monocytes, macrophages and endothelial cells), and LTD_4_ (another eicosanoid inflammatory mediator), both of which stimulate Cl^−^ secretion in non-inflamed mammalian colon [15], [17], failed to stimulate KCNQ1/KCNE3 channel activity in control crypts, due to either the acute nature of our experiments, or the non-involvement of KCNQ1/KCNE3 channels in the Cl^−^-secretory effects of IL-1β and LTD_4_.

In summary, we have shown for the first time that cAMP-stimulated KCNQ1/KCNE3 channels reside in the basolateral membrane of healthy human colonic crypt cells, and that their activity is increased in active UC. This builds on our previous studies, which indicated an expanded distribution of apical BK channels, with the virtual disappearance of basolateral IK channels, along the surface-crypt axis in these patients [1], [22]. Thus, it is clear that different types of K^+^ channel, each with a crucial role to play in ion transport across healthy human colon, undergo profound changes in expression and activity in active UC. A more detailed picture is therefore emerging about the pathophysiology of diarrhoea in this disease [1]. Given the recent evidence in human colon for partitioning of Cl^−^ and K^+^ secretory pathways between colonocytes and goblet cells respectively [32], further studies are required to map different types of K^+^ channel along the surface-crypt axis in healthy and inflamed colon, and to identify the intracellular mechanisms that modify K^+^ channel expression during the inflammatory process.

## Conflict of interest

None of the authors have any actual or potential conflicts of interest.

## Author contribution statement

JEL, MH and GIS conceived the experiments and analysed the data. AA-H, JEL and QA carried out the experiments, and acquired and analysed the data. GIS and MH were involved in writing the paper. All authors approved the submitted version of the paper.
